# Oxlignin: A Novel Type of Technical Lignin from Kraft Pulp Mills

**DOI:** 10.1021/acsomega.5c00434

**Published:** 2025-04-28

**Authors:** Jenny Sjöström, Louise Brandt, Gunnar Henriksson, Olena Sevastyanova

**Affiliations:** † Department of Fiber and Polymer Technology, School of Engineering Sciences in Chemistry, Biotechnology and Health, Royal Institute of Technology, KTH, Teknikringen 56-58, Stockholm 114 28, Sweden; ‡ Wallenberg Wood Science Center (WWSC), Department of Fiber and Polymer Technology, School of Engineering Sciences in Chemistry, Biotechnology and Health, Royal Institute of Technology, KTH, Teknikringen 56-58, Stockholm 114 28, Sweden

## Abstract

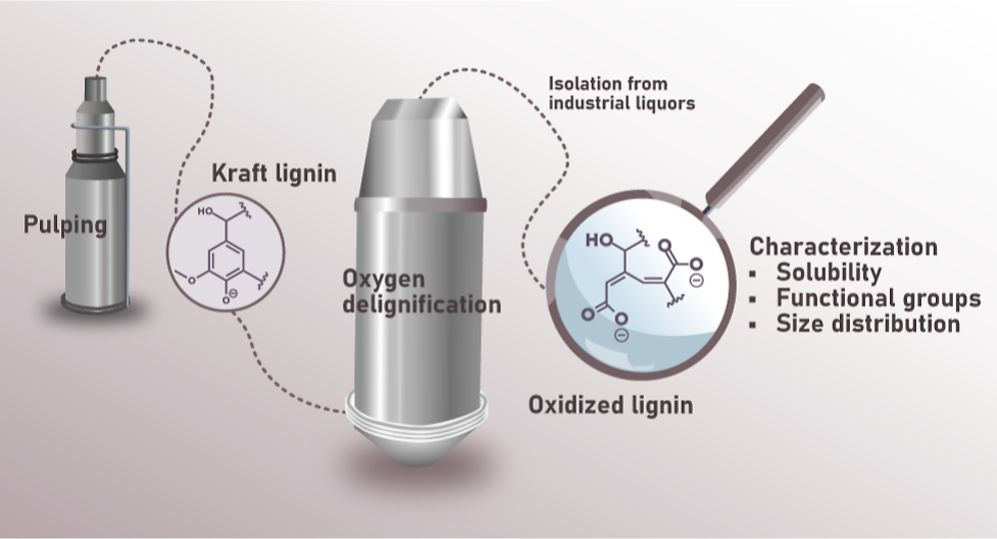

Lignin, a bio-originated polymer, is being explored as an alternative to nonrenewable fossil resources. It is obtained from biomass during pulping and is mostly burned for energy. In most kraft pulp lines, residual lignin in the pulp is oxidized and solubilized during an oxygen delignification step. This study proposes an isolation method for lignin solubilized during oxygen delignification, which we refer to as “oxlignin”, and explores its structural characteristics and properties. The study found acid precipitation to be an effective method for partially isolating oxlignin from the oxygen delignification step. Various analytical methods were employed, including UV–vis absorption analysis, ^31^P NMR spectroscopy, FT-IR spectroscopy, SEC, and TGA. In addition, the solubility of the lignin was studied in four different solvents and compared to the commercial kraft lignins. The study found that oxlignin is a promising substitute for lignosulfonates in certain applications due to its hydrophilicity and high solubility in water, methanol, and ethanol. Compared to kraft lignins, oxlignin has a lower phenolic group content but higher carboxylic acid content.

## Introduction

For many years, kraft pulping has been the largest process for turning wood into paper grade and dissolving pulp.^1^ Most of the lignin in wood is broken down into monomers/oligomers during this process. It undergoes a series of reactions that result in strongly colored oligomers soluble in strong alkaline solutions.^2^ During pulping, carbohydrate degradation products are also formed. Some of these molecules can also exhibit strong color in the liquor, giving it a dark color and the name black liquor. After separating the produced pulp, the black liquor undergoes a multistep chemical recovery system that produces pulping chemicals (white liquor) and excess energy.^3,4^ However, the equipment for this process is expensive and can be a limiting factor in pulp mills.^5^

In recent years, there has been significant interest in extracting part of the lignin degradation products from black liquor. Such kraft lignins, which are useable as solid fuel, have gained a lot of interest in various types of applications.^6−9^ Several methods have been developed for extracting lignin from black liquor with the Westvaco method as the first commercialized process.^10^ This method is based on different forms of precipitations based on the low solubility of kraft lignin at neutral or acidic pH.^8^

The structure of kraft lignin is complex due to a series of reactions in the pulping liquor. More studies are, however, needed to unveil the technical potential of oxlignin. Understanding its behavior and interactions in various applications will be crucial for optimizing its use as a sustainable alternative in different industries. Future research should focus on the scalability, cost-effectiveness, and long-term performance of oxlignin in practical applications. Additionally, exploring its compatibility with other materials and its effects on product formulations will further enhance its utility in various sectors, including construction, agriculture, and bioplastics. By addressing these areas, we can better assess the viability of oxlignin as a renewable resource in the biorefinery landscape,^11,12^ and it may also contain material of carbohydrate origin^13^ and covalently bound sulfur.^14^ This complexity of kraft lignin makes it difficult to use for certain applications. The most commercially utilized lignin, however, is not kraft lignin but lignosulfonates.^15,16^ Lignosulfonates are recovered from the sulfite process and have been used in a variety of applications for many years.^17−19^ Lignosulfonates differ from kraft lignin by exhibiting high solubility in a wide pH range, yet carrying hydrophobic/aromatic functionality. Lignosulfonates are therefore useable as, for example, dispersing agents for concrete and asphalt.^17^ It is important to note that while the sulfite process is still used for pulp production, its usage has declined over time, and it now only accounts for a small percentage of the total annual pulp production.^1^

Thus, it would be interesting if a technical lignin with properties similar to those of lignosulfonate could be prepared from kraft lignin. Westvaco kraft lignin has indeed been sulfonated,^20^ but another possibility is to create carboxylic acids through oxidation reactions; an alkali-O_2_ oxidation method has been presented for creating lignin dispersants in a kraft pulp mill. By utilizing O_2_ gas to enhance the anionic charge of kraft lignin, the resulting product can be used as a versatile dispersant or concrete plasticizer.^21^ Research conducted has demonstrated that this oxidized lignin substance surpasses lignosulfonates and is equally effective as synthetic materials in its ability to serve as a plasticizer and dispersant agent.^22,23^ Furthermore, a growing body of literature recognizes the potential of oxidized lignin from different sources and in different applications.^24,25^ Recent research has explored the oxidative valorization of lignin, demonstrating its potential to produce high-value chemicals such as vanillin, syringaldehyde,^26^ and phenolic monomers.^27,28^ Studies have highlighted the effectiveness of kraft delignification in recovering lignin and polysaccharides from eucalyptus processing,^29^ as well as the optimization of reaction conditions to enhance the yield and quality of lignin-derived products.^30^

**Figure 1:**
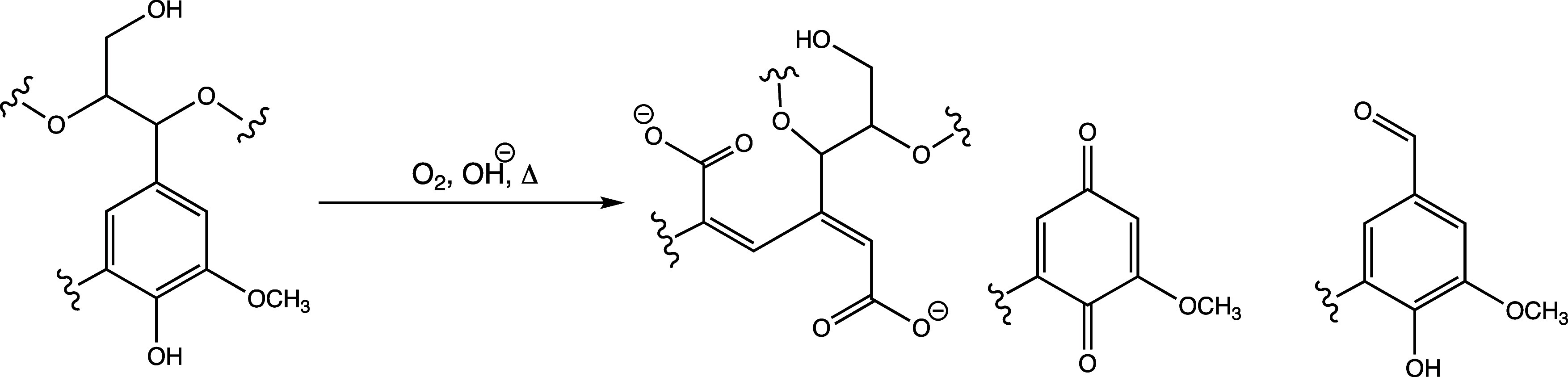
Example of structural changes in lignin during oxygen delignification.

**Figure 2:**
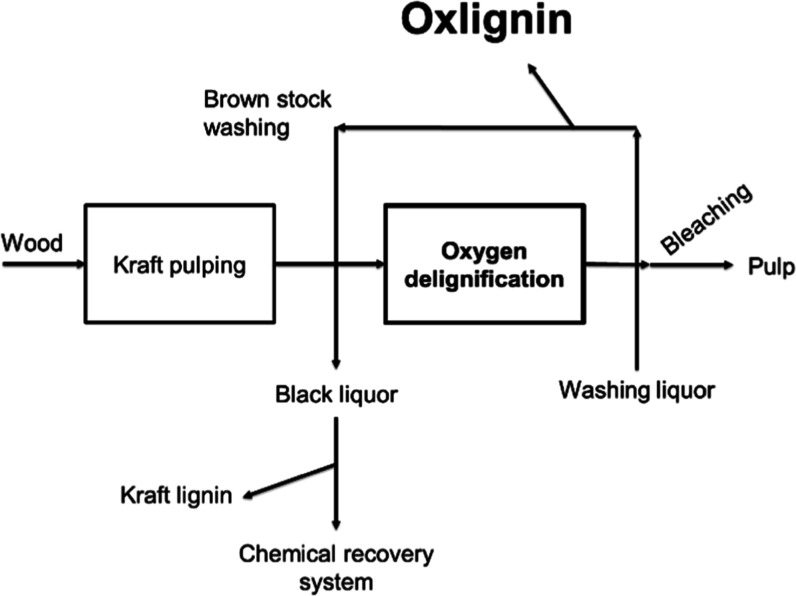
Schematic representation of the various processes involved in a kraft mill, along with the products that can be extracted.

Another way of obtaining oxidized lignin could be to utilize the material released during the oxygen delignification process, herein named oxlignin. Oxygen delignification is usually carried out on kraft pulp to eliminate most of the remaining lignin present in kraft pulp.^31−35^ During oxygen delignification in the kraft pulp mill, oxygen reacts with the remaining lignin in the pulp, which leads to fragmentation and charge introduction, thereby solubilizing the lignin. High pressure, temperature, and pH are applied to compensate for the low solubility and weak oxidative ability of oxygen.^36^ The oxidation of free phenolic hydroxyl groups entails the formation of resonance-stabilized phenoxy radicals.^37,38^ Common products in these reactions are highly soluble muconic acid (Figure 1), quinones, and vanillin-like structures.^31,37−40^ The resulting liquids from washing the extracted lignin from the pulp are typically utilized in brown stock washing, which indicates that kraft lignin can be partly derived from this process, as shown in Figure 2.

Extracting oxidized lignin directly after an oxygen delignification stage is expected to give a product more homogeneous than that of kraft lignin. The properties are also expected to be different from the main bulk of the kraft lignins, especially the solubility in water, which probably will be higher, particularly at lower pH.

This study focuses on isolating and characterizing partly oxidized lignin, called oxlignin, which is a specific fraction solubilized during the oxygen delignification stage in the kraft pulp production process. The research presents a lab-scale method for preparing oxlignin from industrial oxygen delignification washing liquors and characterizing the material physically and chemically by comparing it to conventional kraft lignins. Characterization of residual lignin from oxygen delignification has been explored in various studies,^41,42^ primarily focusing on controlled laboratory methods. However, our study isolates lignin directly from the oxygen delignification stage of kraft pulping, providing an industrially relevant lignin source. This approach not only offers insights into the characteristics of oxlignin but also explores its potential as a sustainable substitute for lignosulfonates, addressing gaps in previous research.

## Material and Methods

### Materials

Washing liquor from the double oxygen delignification step in the kraft pulp production process using a mix of Scots pine (*Pinus sylvestris*) and Norway spruce (*Picea abies*) was produced at Östrand massafabrik, Sundsvall, Sweden, and kindly provided by SCA. LignoBoost lignin prepared from softwood kraft pulping black liquor made at the Sunila mill, Kotka, Finland was kindly provided by Stora Enso. Indulin, lignin prepared from high kappa kraft pulping of pine, was purchased from Mead-Westvaco (USA). Both the LignoBoost kraft lignin and Indulin AT were used as delivered. All other chemicals were of analytical grade.

### Methods

In this study, a series of tests were conducted to characterize the properties of oxlignin obtained through a precipitation method. The investigation included solubility determination in various solvents, quantification of functional groups using ^31^P NMR spectroscopy, Fourier Transform Infrared (FT-IR) analysis, thermal analysis, proximate analysis, and molecular weight analysis. These analyses aimed at elucidating the properties and composition of oxlignin, providing essential insights into its potential applications and behavior in various environments. The methods employed were built upon the project’s aim to understand the characteristics of oxlignin.

#### Preparation of Oxlignin

**Figure 3:**
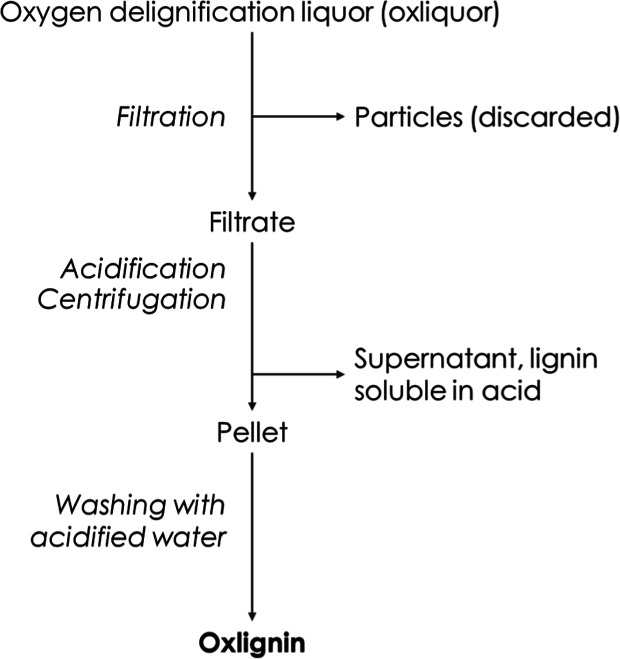
Process of separating and obtaining lignin from the oxygen delignification filtrate.

Oxlignin was prepared by precipitation from washing liquor after an industrial double oxygen delignification stage following^16^ with some modifications. For this, the washing liquor was filtered through a filter paper on a Buchner funnel, and the pH of the filtrate was adjusted to 2 using 72% sulfuric acid. The sample was left overnight at room temperature for precipitation and sedimentation, after which the clear solution was decanted and discarded, and the remaining material was centrifuged at 4200 rpm for 10 min with a Hettich centrifuge. The supernatant was saved for further analysis, and the lignin pellet was resuspended in acidified (pH 2) water and recentrifuged. The pellet, oxlignin, was freeze-dried and used for further investigations. The method is summarized in Figure 3.

#### Solubility in Different Solvents

The solubility of the lignins was determined by adding 1 g of each to 10 mL of the selected solvents (water, methanol, ethanol, and ethyl acetate) before stirring for 2 h. The water sample was adjusted to a neutral pH (pH 6.5) by dissolving the sample in alkaline water (NaOH 0.5 M). The solutions were centrifuged, and the supernatant was collected for dry content measurement. The solubility was calculated based on the dry content of the supernatant and mass of the solvent.

#### Analysis of Oxlignin

##### UV Spectroscopy to Quantify Lignin in the Various Fractions

UV–visual spectra over 250–700 nm were recorded using a UV-2550 spectrophotometer, Shimadzu, Japan. All samples were adjusted to pH 12 before analysis.

The concentration of lignin in the wash liquor was determined using the standard addition method with precipitated lignin as the standard. A standard stock solution was prepared at a concentration of 927 g/L (ash content is considered). Aliquots of wash liquor were transferred into vials, and increasing amounts of the lignin standard solution were added to separate vials to create a range of concentrations. The samples were mixed thoroughly before analysis. Absorbance measurements were performed using a UV-2550 spectrophotometer (Shimadzu, Japan) set at 280 nm. A calibration curve was constructed by plotting the absorbance against the added lignin concentration, and the original lignin concentration in the wash liquor was determined by extrapolating the linear regression to the *x*-axis.

##### NMR-Spectroscopy

The amount of different functional groups in the isolated lignin was investigated using ^31^P NMR spectroscopy.^31,43^ A solution was prepared by adding 30 mg of lignin to a mixture of 100 μL of pyridine and 100 μL of *N*,*N*-dimethylformamide, which was stirred constantly until the lignin was fully dissolved. Next, 50 μL of *N*-hydroxy-5-norbornene-2,3-dicarboximide (60 mg/mL) was added, and the solution was mixed for an additional 30 min. Following this, 100 μL of the derivatization agent 2-chloro-4,4,5,5-tetramethyl-1,3,2-dioxaphospholane, which had a concentration of 60 mg/mL with 5 mg/mL chromium(III) acetylacetonate in pyridine, was added to the solution. Finally, 450 μL of chloroform-*d* was added, and the mixture was allowed to react for 2 h before being transferred to an NMR tube. The analysis was conducted using a Bruker NMR spectrometer Avance III HD 400 Hz with 256 scans and a delay time of 5 s. The peak integration was annotated using MestReNova software with auto baseline correction, as described elsewhere.^43^

##### FT-IR Spectroscopy

A PerkinElmer Spectrum 2000 FT-IR instrument, which was equipped with an MKII Golden Gate single reflection ATR system, was used for the FT-IR analysis. The spectra were acquired at room temperature between 600 and 4000 cm^–1^ with 16 scans averaged at a 4.0 cm^–1^ resolution. All spectra were analyzed with PerkinElmer Spectrum software, which provided automatic ATR- and baseline correction. To normalize the peaks, the aromatic C–H deformation at the 1030 cm^–1^ peak was used,^44^ and peak analysis was performed according to the literature.^45−47^

##### Thermogravimetric Analysis

Approximately 10 mg sample was placed in a ceramic crucible and inserted into a TGA/DSC 1 instrument (Mettler Toledo), and the analysis was performed according to previously described methods.^48^ The samples were heated in a N_2_ atmosphere with a flow rate of 50 mL/min from 30 to 800 °C (10 °C/min) to determine the amount of char and thermal stability. To determine the resistance to oxidative degradation and ash content, all three samples were heated from 30 to 800 °C at the same rate in an O_2_ atmosphere (50 mL/min). Finally, the STARe excellence software was utilized to calculate the first derivative and subtract the blank from the sample data.

To assess the content of ash, fixed carbon, volatile, and organic matter, a proximate analysis was conducted as previously described.^49^ Weight losses were measured directly under N_2_ and O_2_ atmospheres, with the temperature being raised from 30 to 800 °C in N_2_ and then reduced to 450 °C before being raised again to 800 °C in O_2_. The STARe excellence software was used for data analysis.

##### Size Exclusion Chromatography

An Infinity 1260 instrument (Polymer Standard Services, Germany) equipped with UV and RI detectors was used for molecular weight analysis. The system consists of a PSS 100 Å column, a PSS GRAM 10 000 Å analytical column, and a PSS precolumn. To prepare the samples, 1 mg of lignin was dissolved in 2 mL of eluent, dimethyl sulfoxide (DMSO) with 0.5 wt % lithium bromide (Li–Br). The filtered samples were then transferred to glass vials before analysis. Standard calibration was performed using Pullulan standards. Baseline correction, comparison with the calibration curve, and calculation of molecular weight and polydispersity index were executed by using the software PSS WinGPC.

##### Dispersing Ability

Samples of 30 mg of lignin (oxlignin or lignosulfonate) were dissolved in 5 mL of deionized water. Fifty milligrams of bentonite clay (wine-clearing quality) were dispersed in the lignin solution and a sample of 5 mL of water, as reference. The samples were mixed and left overnight at room temperature. The phases and particles of the samples were inspected regularly.

## Results and Discussion

Oxlignin was prepared from oxygen delignification liquor (Figure 3) by pH precipitation. Its properties were compared with those of Indulin AT and Lignoboost lignin. The study first examined the solubility of oxlignin, LignoBoost, and Indulin AT lignins in methanol, ethanol, ethyl acetate, and water, finding oxlignin to be highly soluble in polar solvents. Thermal properties were then assessed through thermogravimetric analysis, revealing oxlignin’s lower thermal stability and higher ash content. The chemical structure of the lignins was analyzed using ^31^P NMR, which showed higher carboxylic acid content and fewer phenolic groups in oxlignin. FT-IR spectroscopy further identified structural differences, while UV-spectroscopy and size exclusion chromatography provided insights into oxlignin’s unique spectral features and more homogeneous molecular weight distribution.

### Characterization of the Properties of Oxlignin

#### Solubility

**Figure 4:**
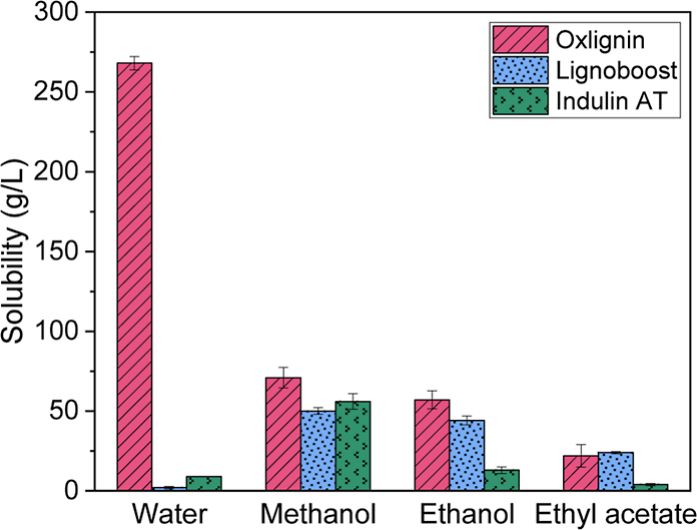
Solubility in g/L of oxlignin, LignoBoost lignin, and Indulin in water, methanol, ethanol, and ethyl acetate. Samples were analyzed in duplicates.

The solubility of three types of lignin materials was tested in four different solvents, namely, methanol, ethanol, ethyl acetate, and water, and their solubility was compared with each other. The results are shown in Figure 4.

The oxlignin showed high solubility properties in water, whereas the solubility of the kraft lignins was very low. Oxlignin also exhibits the highest solubility in methanol and ethanol among the three lignin materials. However, LignoBoost has the highest solubility in ethyl acetate. All lignins were fully dissolved to the reported concentrations without noticeable fractionation. This indicates that oxlignin has a much more polar and hydrophilic structure than kraft lignin and opens up to similar applications as lignosulfonates and oxidized kraft lignin such as plasticizers and dispersing agents (Aro and Fatehi, 2017).^17^ Due to the higher solubility, oxlignin demonstrated strong potential to be used in sustainable polyurethane coatings.^50^

#### Thermal Properties

**Figure 5:**
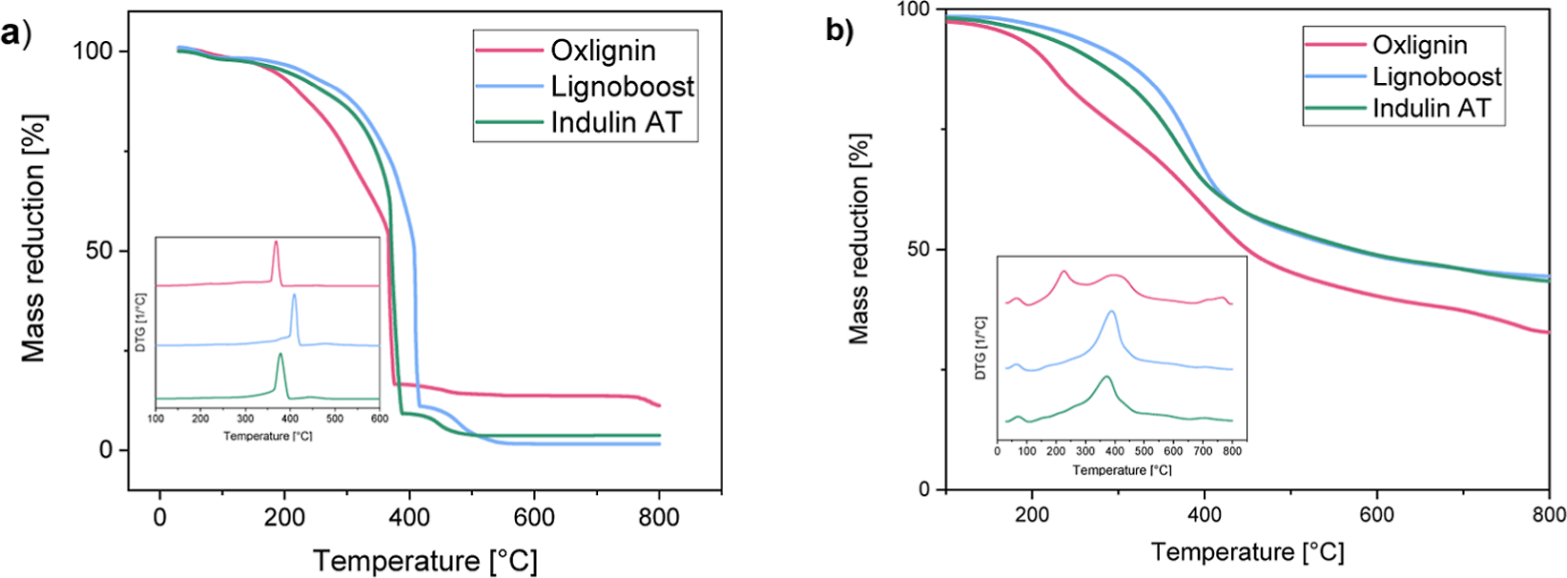
(a)TGA curves in the O_2_ environment and (b) N_2_ environment for oxlignin, LignoBoost lignin, and Indulin AT lignin. The corresponding DTG graphs are shown in the figures.

An analysis was conducted on the thermal properties of oxlignin and the reference lignin materials through thermogravimetric analysis (TGA). The samples were analyzed in both N_2_ gas flow to measure thermal stability and in the O_2_ gas flow to measure resistance to oxidative degradation, as seen in Figure 5a,b, respectively.

From the oxidative analysis, it is apparent that the ash content is higher for oxlignin (11.2%) compared to LignoBoost lignin (1.5%) and Indulin AT lignin (3.8%), which could be due to insufficient washing of salts during the washing procedure of the isolation process or due to intrinsic properties of the material. Further optimization of the washing process or additional purification steps may be needed to reduce these impurities. Thermal stability and resistance to oxidative breakdown are assessed by the temperature at which a 5% weight loss is observed. Analysis of the first derivative of degradation curves reveals shifts in the degradation rate and the temperature corresponding to the maximum degradation rate. These metrics, namely, thermal stability and the temperature of maximum degradation, are summarized in Table 1.

**Table 1:** Thermal Stability of Oxlignin, LignoBoost Lignin, and Indulin AT Lignin^a^

	O_2_ flow		N_2_ flow	
	*T*_5%_ [°C]	*T*_max_ [°C]	*T*_5%_ [°C]	*T*_max_ [°C]
Oxlignin	182	370	170	227
LignoBoost	228	409	237	390
Indulin AT	200	380	203	370

a *T*_5%_ corresponds to the temperature where 5% of the original sample weight has been degraded. *T*_max_ corresponds to the temperature where the maximum degradation rate has been reached.

The temperature at which 5% weight loss occurs in a nitrogen environment shows some distinctions among the materials, indicating that oxlignin has a lower thermal stability. Similarly, the susceptibility to oxidative breakdown, assessed through the temperature of 5% degradation in an oxygen atmosphere, exhibits variability, with oxlignin displaying the lowest stability. The temperature of maximum degradation, determined as the temperature corresponding to the first derivative’s maximum point, notably varies across the lignin samples in nitrogen environment. LignoBoost and Indulin AT curves in a nitrogen flow exhibit singular distinct minima, while oxlignin presents multiple peaks. This signifies a more heterogeneous material concerning thermal stability with distinct structures volatilizing at varying temperatures. However, in oxygenated flow, there is a smaller difference between oxlignin and the reference samples.

### Structure Characterization

To investigate which kinds of structures oxlignin possesses that give the above-described properties, various structural characterizations were performed, and the results were compared to the kraft lignins: LignoBoostLignoBoost and Indulin AT.

#### ^31^P NMR

**Figure 6:**
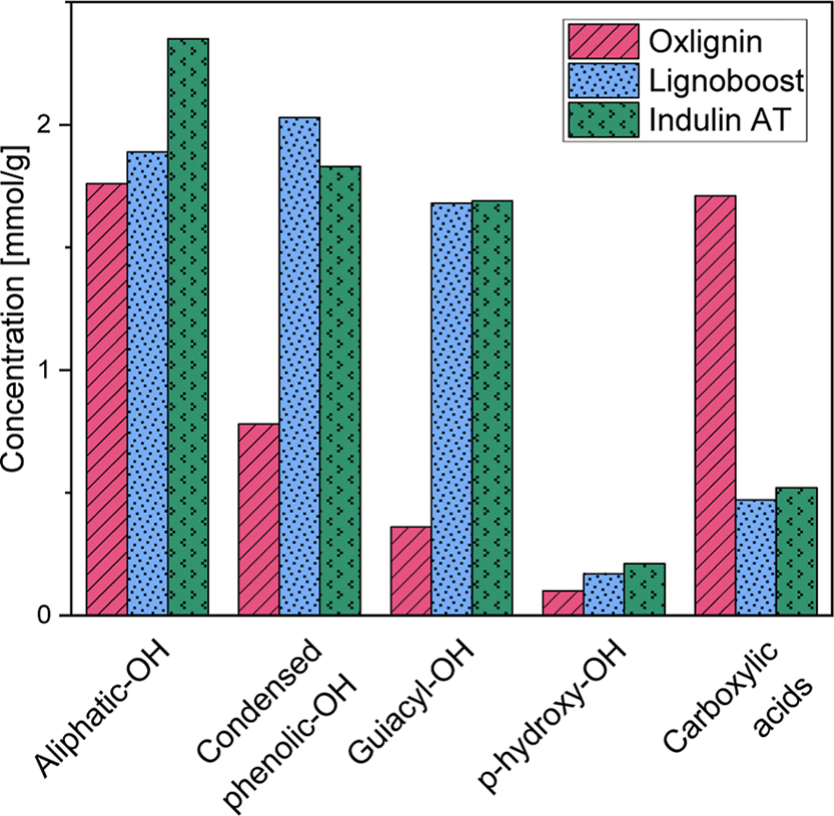
Concentrations of functional groups in mmol/g lignin in oxlignin, LignoBoost, and Indulin based on ^31^P NMR spectroscopy.

Figure 6 displays the results obtained from ^31^P NMR spectroscopy, which were performed to investigate the structure of the oxlignin and the reference lignin samples. The hydroxyl groups in lignin were analyzed quantitatively after substitution with phosphorus-containing species. The functional groups that were analyzed include aliphatic hydroxyl groups, condensed phenolics, guaiacyl units, *p*-hydroxyphenyl units, and carboxylic acids.

Oxlignin has a significantly higher number of carboxylic acids in comparison to LignoBoost and Indulin. This result is aligned with previous studies.^35^ However, the total phenolic hydroxyl groups are lower in oxlignin due to the lower amounts of condensed phenolics and guaiacyl units. These findings support the theory of the formation of muconic acid resulting from the ring opening of aromatic structures (Figure 1). Despite a smaller difference in the amount of aliphatic hydroxyl groups between oxlignin and LignoBoost compared to Indulin, this difference could be due to the lesser degradation of aliphatic structures in Indulin, which spends less time in the cooking liquor.

#### FT-IR Spectroscopy Data

**Figure 7:**
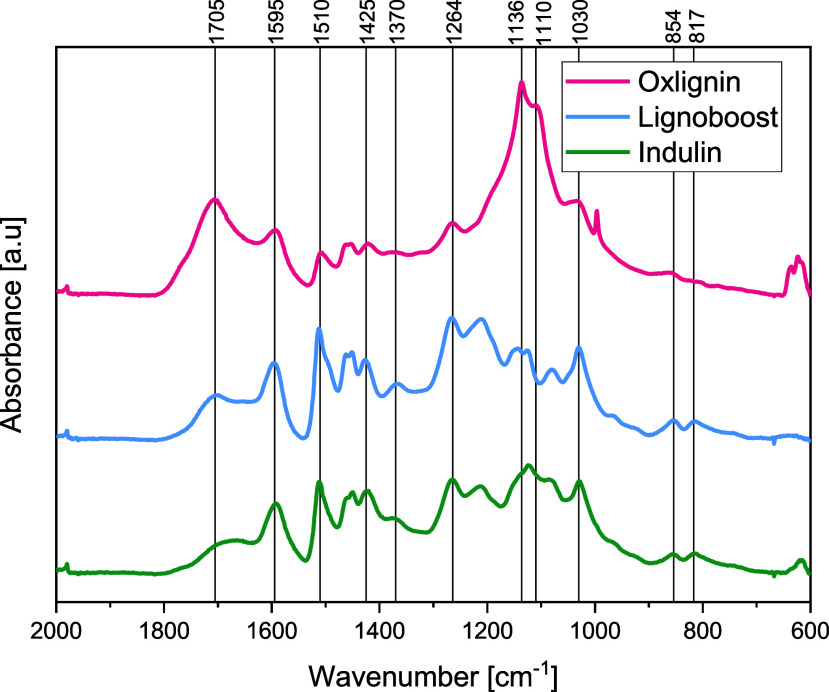
FT-IR spectra of oxlignin, LignoBoost lignin, and Indulin in the range of 600–2000 cm^–1^.

An analysis of the chemical composition of the samples using Fourier-transform infrared spectroscopy was conducted. The data in Figure 7 were obtained through the acquisition of FT-IR spectra in the range of 600–2000 cm^–1^, which allowed us to study the molecular vibrations and structures of the samples. Table S1 (Supporting Information) presents the structure corresponding to the assigned peaks.^45^

Most of the peaks in all three spectra occur at similar wavenumbers but with varying intensities. In the fingerprint region, there are distinct differences in wavenumbers, but due to the complexity of this region for most compounds, it is challenging to attribute peaks to specific structures. Additionally, the intensity of the peaks is normalized based on the peak at 1030 cm^–1^, which is known to correlate with C–O deformation in primary alcohols, according to the literature. Since it is uncertain whether the number of these bonds remains constant during the oxygen delignification, it is not possible to directly compare the intensities of the different spectra. However, the relationship between the intensity of the normalization peak and other peaks can be discussed.

All three lignins exhibit a broad band in the range of 3700–3000 cm^–1^, indicating phenolic and aliphatic hydroxyl groups. Compared to the normalization peak, LignoBoost and Indulin AT have a more broad peak than oxlignin. The peaks at 1510, 1425, 1370, 1264, 854, and 817 cm^–1^ correspond to the structures associated with aromatic rings and are more intense relative to the normalization peak in LignoBoost and Indulin AT compared to oxlignin. This suggests that the number of phenolic structures is lower in the oxlignin than that in the kraft lignins. The peak at 1705 cm^–1^ is one of the peaks that is higher in oxlignin relative to the normalization peak. This peak is associated with conjugated aldehydes and carboxylic acids, which supports the theory of muconic acid formation during oxygen delignification. The bands at 1140 and 1128–1125 cm^–1^ in the kraft lignin samples overlap with each other but have fairly low intensity. The 1136 band is the most intense band in the oxlignin spectra, indicating a difference in the structure compared to the kraft lignins. If this peak originates from lignin structures, it corresponds to aromatic C–H in-plane deformation, secondary alcohols, or C=O stretches. However, this peak could also originate from other uncharacterized structures. Further analysis is required to determine the originating structure of this peak. The peak at 1110 cm^–1^ corresponds to the aromatic C–H deformation of S units.^46^ However, the pulp mill where the liquor was collected used softwood, which does not contain syringyl units. Therefore, this peak presumably originates from uncharacterized structures, and further analysis is needed to determine any conclusions.

#### UV-Spectroscopy

**Figure 8:**
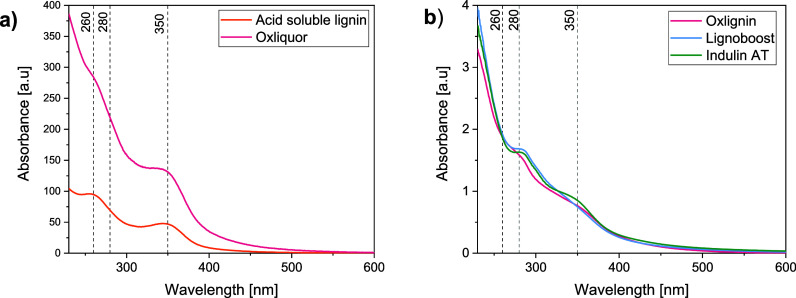
(a) UV–vis absorption spectra of the soluble lignin after acidification and the starting material, oxyliquor, as well as (b) three dissolved lignin samples (oxlignin, LignoBoost lignin, and Indulin AT).

The UV–vis absorption analysis was utilized to examine the structure of the acid-soluble lignin, which remained in the solution following the isolation process (Figure 3). The pH levels of the acidified solution containing the acid-soluble lignin were adjusted to 12 to enable comparison with the oxliquor, as well as with the dissolved oxlignin and kraft lignin reference samples, which were also adjusted to pH 12. Figure 8 shows the absorptive curves obtained from this analysis.

The absorption peak at 280 nm, which is a typical feature of most technical lignins, is not present in the spectra of acid-soluble lignin and oxliquor, which might indicate a lower content of phenols, which is in line with both the above results and the assumed reactions in oxygen delignification (Figure 1). However, it is evident in both reference lignins and is distinguishable as a shoulder in oxlignin, indicating the existence of unconjugated phenolic groups. The absorption band at 350 nm is associated with conjugated phenolic structures. Although the band is visible in oxliquor and acid-soluble lignin, it is less distinct in oxlignin, LignoBoost lignin, and Indulin AT. Kraft lignin contains conjugated structures such as enol ethers and stilbenes, which are reactive during the oxygen delignification process (Ljunggren and Johansson, 1990). In contrast, other types of conjugated structures, such as quinones and vanillin, are believed to be products of oxygen delignification reactions (Figure 1).

Due to the larger set of resonance structures, conjugated phenols are more stabilized in their deprotonated form and possess a lower p*K*_a_ value than unconjugated phenols. Consequently, conjugated structures are more likely to retain their charge or require a lower pH to be protonated, making them more water-soluble than unconjugated structures and more likely to remain in the liquid phase during the isolation process as an acid-soluble lignin. Unconjugated structures, on the other hand, are more likely to precipitate, resulting in a shoulder at 280 nm in the oxlignin spectrum. The acid-soluble lignin spectrum shows a distinct band at 260 nm, which is visible as a shoulder in the oxliquor, indicating the presence of other acid-soluble structures that have not yet been identified and characterized.

The concentration of lignin in the industrial wash liquor used for oxlignin isolation was determined to be 8.21 g/L based on the standard addition method; see Figure S1 in Supporting Information This number is, however, based on the liquor collected at one time and would probably fluctuate based on mill performance. The mass extinction coefficient was determined from the slope of the curve and was found to be 20.0 L g^–1^ cm^–1^. This value falls within the range of kraft lignins, which is approximately 18.7 to 25.^51^

#### Molecular Weight Distribution

**Figure 9:**
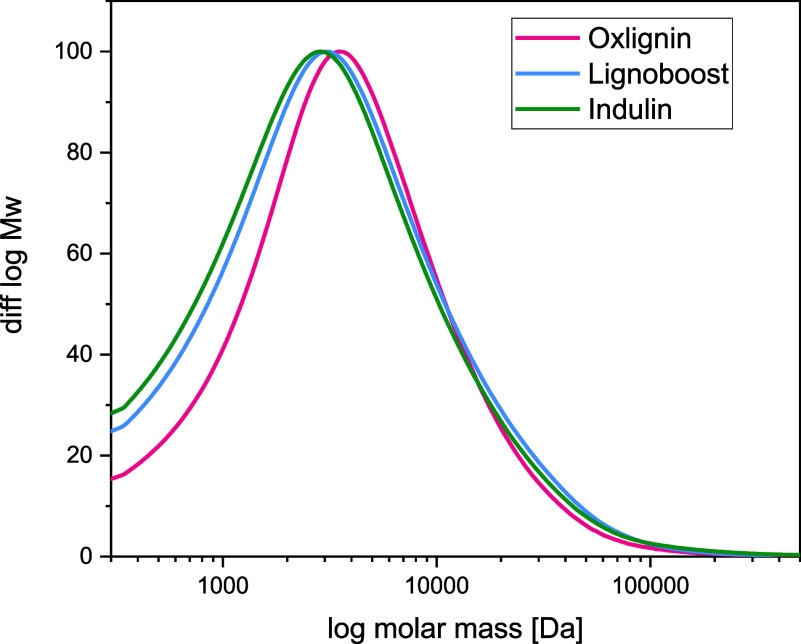
Size exclusion chromatography of the oxlignin, LignoBoost lignin, and Indulin.

Size exclusion chromatography was performed to analyze the average molecular weight and the monodispersity of oxlignin, LignoBoost, and Indulin AT. The elugrams of the three samples are presented in Figure 9.

Since oxlignin probably contains more charged groups than kraft lignin, the hydrodynamic diameter might be slightly different, and the behavior in size exclusion chromatography might therefore differ. With this reservation, the number-average molecular weights , weight-average molecular weights , and the polydispersity indices (PDI) were calculated and are presented in Table 2.

**Table 2:** Number Molecular Weight Distribution, Molar Molecular Weight Distribution, and Polydispersity Index From Size Exclusion Chromatography Analysis of the Three Lignin Samples, Oxlignin, LignoBoost Lignin, and Indulin

sample	(kDa)	(kDa)	PDI
Oxlignin	1.4	5.1	3.8
LignoBoost lignin	0.9	5.6	5.9
Indulin AT	0.9	5.2	5.9

The molecular weight distribution of oxlignin is similar to that of commercial kraft lignins, which may be attributed to the origin of the oxidized lignin from non-dissolved residual kraft lignin present during the oxygen delignification process. The apparent number-average molecular weight of oxlignin is higher than the value for LignoBoost lignin and Indulin AT, while the average molecular weight is somewhat lower. This gives a lower polydispersity index for oxlignin, which means that the molecular weight of the polymers in the sample is more narrowly distributed. These results indicate a higher homogeneity of oxlignin with regard to particle size compared to LignoBoost lignin and Indulin AT.

#### Dispersing Ability

**Figure 10:**
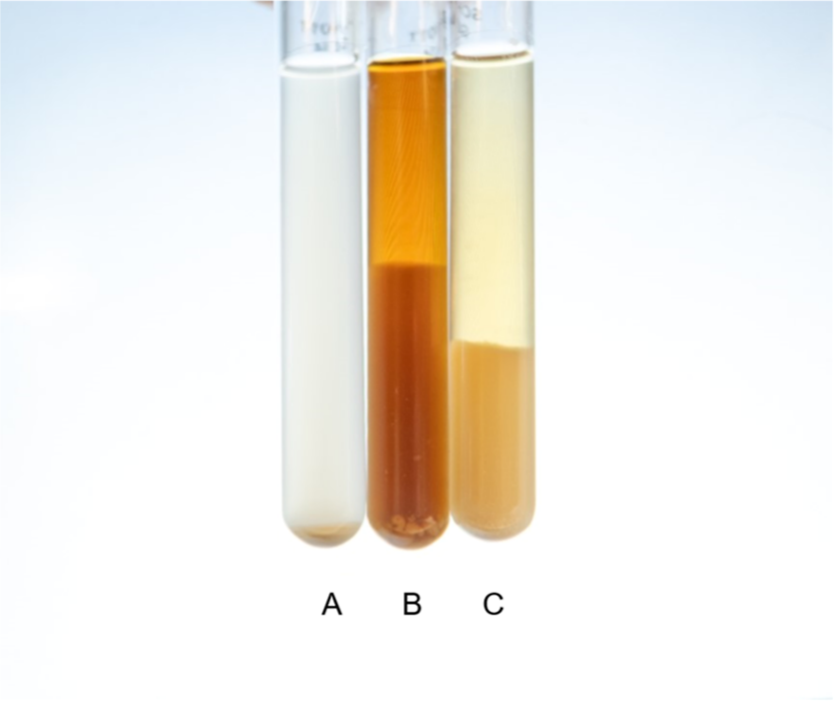
Phase stability of bentonite suspensions with oxlignin and lignosulfonate. Bentonite was dispersed in water (A) or with either oxlignin (B) or a commercial lignosulfonate (C), and phase separation was observed. Photograph courtesy of Carl Moser. Copyright 2025.

The dispersing ability of oxlignin was compared with that of a commercial lignosulfonate for its ability to form a stable phase with bentonite. Bentonite has previously been used to assess the dispersing ability.^52^Figure 10 shows that both lignosulfonate and oxlignin formed a stable phase with bentonite along with a clear water phase, whereas the control without lignin did not. This suggests that oxlignin may have similar dispersing properties as lignosulfonates.

## Conclusions

The proposed isolation method is suitable for the partial isolation of lignin from oxlours, although some lignin remains in the liquid phase as acid-soluble lignin. The structural and solubility characteristics of this acid-soluble lignin differ from those of oxlignin. A noteworthy disparity between oxlignin and the other two lignin materials, LignoBoost lignin and Indulin AT, is the lower presence of phenolic hydroxyl groups in oxlignin. Conversely, oxlignin exhibits a higher carboxylic acid content than the other two materials and possesses the lowest polydispersity index among the three. In terms of solubility, oxlignin demonstrates significantly higher solubility in water than kraft lignins as well as the highest solubility in methanol and ethanol. Also, the ash content of oxlignin is relatively high. Oxlignin demonstrates dispersing properties comparable to those of commercial lignosulfonates and holds potential as an alternative feedstock for applications requiring oxidized lignin. More studies are needed to explore the technical potential of oxlignin. Future research should focus on scalability, cost-effectiveness, and long-term performance of oxlignin in practical applications.

## Supporting Information Available

Structures identified through FT-IR spectroscopy and their assigned wavenumbers and slope graph used to determine the mass extinction coefficient of oxlignin and calculate the initial lignin concentration in the industrial wash liquor (PDF)

## References

[ref1] Mboowa D. A review of the traditional pulping methods and the recent improvements in the pulping processes. Biomass Convers. Biorefin. 2024, 14 (1), 1–12. 10.1007/s13399-020-01243-6.

[ref2] Gierer J. Chemical aspects of kraft pulping. Wood Sci. Technol. 1980, 14 (4), 241–266. 10.1007/BF00383453.

[ref3] Sixta H.; Potthast A.; Krotschek A. W. Chemical pulping processes. In Handbook of Pulp, 2006; . 10.1002/9783527619887.

[ref4] Ulmgren P. Non-process elements in a bleached kraft pulp mill with a high degree of system closure - state of the art. Nord. Pulp Pap. Res. J. 1997, 12 (1), 32–41. 10.3183/npprj-1997-12-01-p032-041.

[ref5] Holmqvist A.; Wallberg O.; Jönsson A. S. Ultrafiltration of Kraft Black Liquor from Two Swedish Pulp Mills. Chem. Eng. Res. Des. 2005, 83 (8), 994–999. 10.1205/cherd.04204.

[ref6] Kalliola A.; Savolainen A.; Ohra-aho T.; Faccio G.; Tamminen T. Reducing the content of vocs of softwood kraft lignins for material applications. Bioresources 2012, 7 (3), 2871–2882. 10.15376/biores.7.3.2871-2882.

[ref7] Nowak A. P.; Hagberg J.; Leijonmarck S.; Schweinebarth H.; Baker D.; Uhlin A.; Tomani P.; Lindbergh G. Lignin-based carbon fibers for renewable and multifunctional lithium-ion battery electrodes. Holzforschung 2018, 72 (2), 81–90. 10.1515/hf-2017-0044.

[ref8] Dos Santos D. A. S.; Rudnitskaya A.; Evtuguin D. V. Modified kraft lignin for bioremediation applications. J. Environ. Sci. Health, Part A: Toxic/Hazard. Subst. Environ. Eng. 2012, 47 (2), 298–307. 10.1080/10934529.2012.640909. 22242883

[ref9] Dessbesell L.; Paleologou M.; Leitch M.; Pulkki R.; Xu C. Global lignin supply overview and kraft lignin potential as an alternative for petroleum-based polymers. Renewable Sustainable Energy Rev. 2020, 123, 109768 10.1016/j.rser.2020.109768.

[ref10] Kienberger M.; Maitz S.; Pichler T.; Demmelmayer P. Systematic Review on Isolation Processes for Technical Lignin. Processes 2021, 9, 804 10.3390/pr9050804.

[ref11] Giummarella N.; Lindén P. r. A.; Areskogh D.; Lawoko M. Fractional Profiling of Kraft Lignin Structure: Unravelling Insights on Lignin Reaction Mechanisms. ACS Sustainable Chem. Eng. 2020, 8 (2), 1112–1120. 10.1021/acssuschemeng.9b06027.

[ref12] Robert D. R.; Bardet M.; Gellerstedt G.; Lindfors E. L. Structural Changes in Lignin During Kraft Cooking Part 3. On the Structure of Dissolved Lignins. J. Wood Chem. Technol. 1984, 4 (3), 239–263. 10.1080/02773818408070647.

[ref13] Lawoko M.; Henriksson G.; Gellerstedt G. New Method for Quantitative Preparation of Lignin-Carbohydrate Complex from Unbleached Softwood Kraft Pulp: Lignin-Polysaccharide Networks I. Holzforschung 2003, 57 (1), 69–74. 10.1515/HF.2003.011.

[ref14] Vishtal A.; Kraslawski A. Challenges in industrial applications of technical lignins. BioResources 2011, 6 (3), 3547–3568. 10.15376/biores.6.3.vishtal.

[ref15] Norgren M.; Edlund H. Lignin: Recent advances and emerging applications. Curr. Opin. Colloid Interface Sci. 2014, 19 (5), 409–416. 10.1016/j.cocis.2014.08.004.

[ref16] Li T.; Takkellapati S. The current and emerging sources of technical lignins and their applications. Biofuels, Bioprod. Biorefin. 2018, 12 (5), 756–787. 10.1002/bbb.1913. PMC6134873 30220952

[ref17] Aro T.; Fatehi P. Production and Application of Lignosulfonates and Sulfonated Lignin. ChemSusChem 2017, 10 (9), 1861–1877. 10.1002/cssc.201700082. 28253428

[ref18] Breilly D.; Fadlallah S.; Froidevaux V.; Colas A.; Allais F. Origin and industrial applications of lignosulfonates with a focus on their use as superplasticizers in concrete. Constr. Build. Mater. 2021, 301, 124065 10.1016/j.conbuildmat.2021.124065.

[ref19] Lora J. Chapter 10 - Industrial Commercial Lignins: Sources, Properties and Applications. In Monomers, Polymers and Composites from Renewable Resources; Belgacem M. N., Gandini A., Eds.; Elsevier, 2008; pp 225–241.

[ref20] Konduri M. K. R.; Fatehi P. Production of Water-Soluble Hardwood Kraft Lignin via Sulfomethylation Using Formaldehyde and Sodium Sulfite. ACS Sustainable Chem. Eng. 2015, 3 (6), 1172–1182. 10.1021/acssuschemeng.5b00098.

[ref21] Kalliola A.; Kangas P.; Winberg I.; Vehmas T.; Kyllönen H.; Heikkinen J.; Poukka O.; Kemppainen K.; Sjögård P.; Pehu-Lehtonen L.; et al. Oxidation process concept to produce lignin dispersants at a kraft pulp mill. Nord. Pulp Pap. Res. J. 2022, 37 (2), 394–404. 10.1515/npprj-2022-0017.

[ref22] Kalliola A. K.; Vehmas T.; Liitiä T.; Tamminen T. LigniOx lignins: High performance concrete plasticizers and versatile dispersants. In NWBC 2018: Proceedings of the 8th Nordic Wood Biorefinery Conference; VTT Technical Research Centre of Finland: Helsinki, Finland, 2018.

[ref23] Vikman M.; Fearon O.; Kalliola A. Biodegradation of Alkali-O2 Oxidized Lignins Used as Dispersants. Bioresources 2022, 17 (4), 6079–6093. 10.15376/biores.17.4.6079-6093.

[ref24] Chatel G.; Rogers R. D. Review: Oxidation of lignin using ionic liquids-an innovative strategy to produce renewable chemicals. ACS Sustainable Chem. Eng. 2014, 2 (3), 322–339. 10.1021/sc4004086.

[ref25] Ma R.; Guo M.; Zhang X. Recent advances in oxidative valorization of lignin. Catal. Today 2018, 302, 50–60. 10.1016/j.cattod.2017.05.101.

[ref26] Pinto P. C. R.; Costa C. E.; Rodrigues A. E. Oxidation of Lignin from Eucalyptus globulus Pulping Liquors to Produce Syringaldehyde and Vanillin. Ind. Eng. Chem. Res. 2013, 52 (12), 4421–4428. 10.1021/ie303349j.

[ref27] Abdelaziz O. Y.; Clemmensen I.; Meier S.; Costa C. A. E.; Rodrigues A. E.; Hulteberg C. P.; Riisager A. On the Oxidative Valorization of Lignin to High-Value Chemicals: A Critical Review of Opportunities and Challenges. ChemSusChem 2022, 15 (20), e202201232 10.1002/cssc.202201232. 36004569 PMC9825943

[ref28] Costa C. A. E.; Vega-Aguilar C. A.; Rodrigues A. E. Added-Value Chemicals from Lignin Oxidation. Molecules 2021, 26 (15), 4602 10.3390/molecules26154602. 34361756 PMC8346967

[ref29] Pinto P. C. R.; Oliveira C.; Costa C. A. E.; Rodrigues A. E. Performance of Side-Streams from Eucalyptus Processing as Sources of Polysaccharides and Lignins by Kraft Delignification. Ind. Eng. Chem. Res. 2016, 55 (2), 516–526. 10.1021/acs.iecr.5b03712.

[ref30] Pinto P. C. R.; Oliveira C.; Costa C. A.; Gaspar A.; Faria T.; Ataíde J.; Rodrigues A. E. Kraft delignification of energy crops in view of pulp production and lignin valorization. Ind. Crops Prod. 2015, 71, 153–162. 10.1016/j.indcrop.2015.03.069.

[ref31] Asgari F.; Argyropoulos D. S. Fundamentals of o×ygen delignification. Part II. Functional group formation/elimination in residual kraft lignin. Can. J. Chem. 1998, 76 (11), 1606–1615. 10.1139/cjc-76-11-1606.

[ref32] Hsu C. L.; Hsieh J. S. Reaction kinetics in oxygen bleaching. AIChE J. 1988, 34 (1), 116–122. 10.1002/aic.690340113.

[ref33] van Heiningen A. R. P.; Ji Y.; Jafari V. Recent Progress on Oxygen Delignification of Softwood Kraft Pulp. In Cellulose Science and Technology, 2018; pp 67–97.

[ref34] Graztl J. S.; Nakano J.; Singh R. P.; ; ; ; North Carolina State University School of Forest Resources Technical Association of the Pulp and Paper Industry Pulp Bleaching Committee. In Chemistry of Delignification with Oxygen, Ozone and Peroxides: 1st International Symposium: Papers; UMI Books on Demand, 1987.

[ref35] Yang R. M.; Lucia L.; Ragauskas A. J.; Jameel H. Oxygen delignification chemistry and its impact on pulp fibers. J. Wood Chem. Technol. 2003, 23 (1), 13–29. 10.1081/WCT-120018613.

[ref36] Käyhkö J.; Peltonen K.; Mutikainen H.; Kopra R.; Eloranta H.; Pesonen A.; Van Heiningen A. The role of gas dispersion in the oxygen delignification process. Tappi J. 2021, 20 (5), 321–328. 10.32964/TJ20.5.321.

[ref37] Gierer J.; Imsgard F. The reactions of lignins with oxygen and hydrogen peroxide in alkaline media. Svensk Papperstidning-nordisk Cellulosa 1977, 80, 510–518.

[ref38] Gierer J.; Reitberger T.; Yang E.; Yoon B.-H. Formation an involvement of radicals in oxygen delignification studied by the autoxidation of lignin and carbohydrate model compounds. J. Wood Chem. Technol. 2001, 21, 313–341. 10.1081/WCT-100108329.

[ref39] Kalliola A.; Kuitunen S.; Liitiä T.; Rovio S.; Ohra-aho T.; Vuorinen T.; Tamminen T. Lignin oxidation mechanisms under oxygen delignification conditions. Part 1. Results from direct analyses. Holzforschung 2011, 65 (4), 567–574. 10.1515/hf.2011.101.

[ref40] Gierer J. Formation and involvement of superoxide (O2-/HO2·) and hydroxyl (OH·) radicals. In TCF bleaching processes: A review. 1997.

[ref41] Moe S. T.; Ragauskas A. J. Oxygen Delignification of High-Yield Kraft Pulp. Part I: Structural Properties of Residual Lignins. Holzforschung 1999, 53 (4), 416–422. 10.1515/HF.1999.069.

[ref42] Gellerstedt G.; Gustafsson K.; Lindfors E. L. Structural changes in lignin during oxygen bleaching; Paper dedicated to Prof. Karl Kratzl on the occasion of his 70th birthday. Nord. Pulp Pap. Res. J. 1986, 1 (3), 14–17. 10.3183/npprj-1986-01-03-p014-017.

[ref43] Meng X.; Crestini C.; Ben H.; Hao N.; Pu Y.; Ragauskas A. J.; Argyropoulos D. S. Determination of hydroxyl groups in biorefinery resources via quantitative (31)P NMR spectroscopy. Nat. Protoc. 2019, 14 (9), 2627–2647. 10.1038/s41596-019-0191-1. 31391578

[ref44] Horikawa Y.; Hirano S.; Mihashi A.; Kobayashi Y.; Zhai S.; Sugiyama J. Prediction of Lignin Contents from Infrared Spectroscopy: Chemical Digestion and Lignin/Biomass Ratios of Cryptomeria japonica. Appl. Biochem. Biotechnol. 2019, 188 (4), 1066–1076. 10.1007/s12010-019-02965-8. 30783948

[ref45] Boeriu C.; Bravo D.; Gosselink R.; van Dam J. E. G. Characterisation of structure-dependent functional properties of lignin with infrared spectroscopy. Ind. Crops Prod. 2004, 20, 205–218. 10.1016/j.indcrop.2004.04.022.

[ref46] Sammons R. J.; Harper D. P.; Labbé N.; Bozell J. J.; Elder T.; Rials T. G. Characterization of organosolv lignins using thermal and FT-IR spectroscopic analysis. Bioresources 2013, 8 (2), 2752–2767. 10.15376/biores.8.2.2752-2767.

[ref47] Faix O. Fourier Transform Infrared Spectroscopy. In Methods in Lignin Chemistry; Lin S. Y., Dence C. W., Eds.; Springer Berlin Heidelberg, 1992; pp 83–109.

[ref48] Gordobil O.; Moriana R.; Zhang L.; Labidi J.; Sevastyanova O. Assesment of technical lignins for uses in biofuels and biomaterials: Structure-related properties, proximate analysis and chemical modification. Ind. Crops Prod. 2016, 83, 155–165. 10.1016/j.indcrop.2015.12.048.

[ref49] Tagami A.; Gioia C.; Lauberts M.; Budnyak T.; Moriana R.; Lindström M. E.; Sevastyanova O. Solvent fractionation of softwood and hardwood kraft lignins for more efficient uses: Compositional, structural, thermal, antioxidant and adsorption properties. Ind. Crops Prod. 2019, 129, 123–134. 10.1016/j.indcrop.2018.11.067.

[ref50] Smyk N. I.; Askari A. S.; Sevastyanova O. Sustainable polyurethane coating with high content of oxylignin isolated from aqueous solution. In 17th European Workshop on Lignocellulosics and Pulp EWLP 2024; Laboratory of Natural Materials Technology; Åbo Akademi University: Turku, Finland, 2024.

[ref51] Dence C. W. The determination of lignin. In Methods in lignin chemistry; Springer, 1992; pp 33–61.

[ref52] Rabaioli M. R.; Miano F.; Lockhart T. P.; Burrafato G. Physical/Chemical Studies on the Surface Interactions of Bentonite With Polymeric Dispersing Agents. In SPE International Symposium on Oilfield Chemistry; SPE-25179-MS; OnePetro, 1993. 10.2118/25179-ms

