# Supplementary material for: Oxlignin: A Novel Type of Technical Lignin from Kraft Pulp Mills
Source: ACS Omega. 2025 Apr 28;10(18):18784–92. doi: 10.1021/acsomega.5c00434 (PMC12079255; doi:10.1021/acsomega.5c00434)
Supplement: Supplementary file 1 — ao5c00434_si_001.pdf [file ao5c00434_si_001.pdf]

Supporting information for:

Oxlignin: A Novel Type of Technical Lignin from Kraft Pulp Mills

Jenny Sjöström [a], Louise Brandt [b], Gunnar Henriksson [a,b] and Olena Sevastyanova [a,b]*

Department of Fiber and Polymer Technology, School for Chemistry, Biotechnology and Health, Royal Institute of Technology, KTH.

Wallenberg Wood Science Center (WWSC), Department of Fiber and Polymer Technology, School for Chemistry, Biotechnology and Health, Royal Institute of Technology, KTH

*Corresponding author E-mail: olena@kth.se

Table S1. The structures identified through FT-IR spectroscopy are presented alongside their respective assigned wavenumbers.

Wavenumber [cm^{-1}]	Structure
**3700-3000**	Phenolic and aliphatic hydroxyl groups
**1705**	Conjugated aldehydes and carboxylic acids
**1595**	Aromatic skeletal vibrations and C=O stretch
**1510**	Aromatic skeletal vibrations
**1425**	Aromatic ring stretching (C-H in-plane deformation)
**1370**	Phenolic OH stretches and aliphatic C-H in methyl groups
**1264**	C-O stretch of guaiacyl unit
**1136**	Aromatic C-H in-plane deformation (1140)/Aromatic C-H in-plane deformation and secondary alcohols and C=O stretch (1128-1125)
**1110**	Aromatic C-H deformation in syringyl units
**1030**	C-O deformation of primary alcohols
**854, 817**	C-H in-plane deformation in guaiacyl

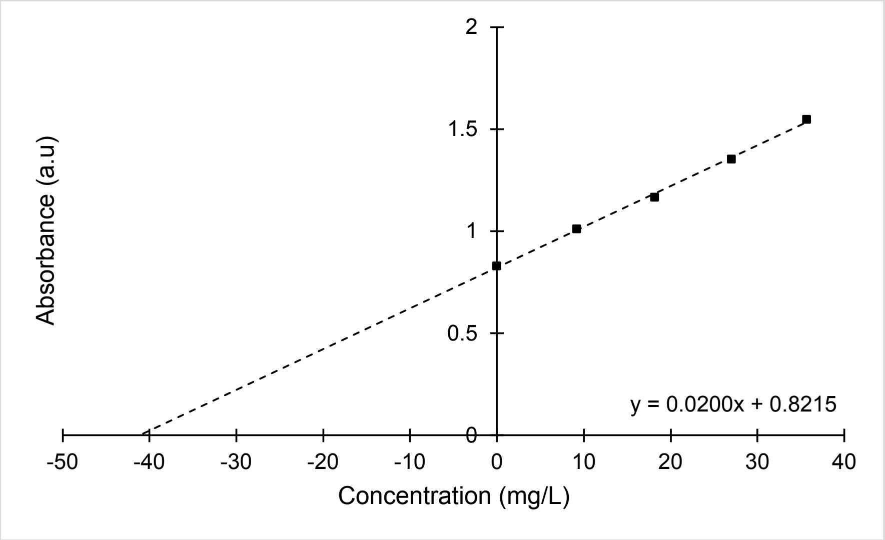

Figure S1. Calibration curve for the standard addition method, used to determine the initial lignin concentration in the wash liquor and calculate the extinction coefficient. The graph plots absorbance at 280 nm against the added lignin concentration, with the x-intercept representing the initial lignin concentration in the sample. The wash liquor was diluted 200 times before measurement. The x-intercept represents the initial lignin concentration in the diluted sample and was multiplied by 200 to obtain the true concentration in the undiluted wash liquor. The linear regression slope was used to determine the mass extinction coefficient.
